# Reaching 100 in the Countryside: Health Profile and Living Circumstances of Portuguese Centenarians from the Beira Interior Region

**DOI:** 10.1155/2018/8450468

**Published:** 2018-06-13

**Authors:** Rosa Marina Afonso, Oscar Ribeiro, Maria Vaz Patto, Marli Loureiro, Manuel Joaquim Loureiro, Miguel Castelo-Branco, Susana Patrício, Sara Alvarinhas, Tatiana Tomáz, Clara Rocha, Ana Margarida Jerónimo, Fátima Gouveia, Ana Paula Amaral

**Affiliations:** ^1^University of Beira Interior, Rua Marquês d'Ávila e Bolama, 6201-001 Covilhã, Portugal; ^2^Center for Health Technology and Services Research (CINTESIS), Faculty of Medicine, University of Porto, Rua Dr. Plácido da Costa, 4200-450 Porto, Portugal; ^3^Abel Salazar Biomedical Sciences Institute, University of Porto, Rua Jorge de Viterbo Ferreira 228, 4050-313 Porto, Portugal; ^4^University of Aveiro, Campus Universitário de Santiago, 3810-193 Aveiro, Portugal; ^5^Health Sciences Research Centre (CICS), Avenida Infante D. Henrique, 6200-506 Covilhã, Portugal; ^6^Cluster of Cova da Beira Health Centers (ACeS Cova da Beira), Av. 25 de Abril, 6200-090 Covilhã, Portugal; ^7^Research Center in Sport Sciences, Health Sciences and Human Development (CIDESD), Quinta de Prados, Edifício de Ciências do Desporto, 5001-801 Vila Real, Portugal

## Abstract

The interest in studying a specific population of centenarians who lives in the country's interior region (PT100-BI) emerged during the first Portuguese systematic study about centenarians (PT100 Oporto Centenarian Study). This region of Portugal is predominantly rural and is one of the regions with the largest number of aged people. The aim of this study is to provide information on the centenarians who live in the Beira Interior region, specifically in terms of their health status and the health services they use. A total of 101 centenarians (mean age: 101.1 years; SD = 1.5 years), 14 males and 87 females, were considered. Most centenarians lived in the community, and 47.6% lived in nursing homes. Nearly half (47.5%) presented cognitive functioning without deficits. A noteworthy percentage presented conditioned mobility and sensory problems. The most common self-reported diseases include urinary incontinence (31.7%), high blood pressure (23.8%), and heart conditions (19.8%). Despite these health and functional characteristics, formal support services and technical assistance were found to be scarcely used. Further research is needed to understand how the role of contextual variables and the countryside environment contribute to the centenarians' adaptation to advanced longevity.

## 1. Introduction

It is estimated that more than 50% of babies born in developed countries since 2000 will live to be more than 100 years old [1]. This is a controversial perspective, however, as it is opposed by several authors who have a less optimistic view [2]. Nonetheless, it is known that a growing number of people in the most developed countries are living up to the age of 100.

Globally, considering the total world population, the estimated number of centenarians in 2013 was 441,000; 3.4 million are expected in 2050, and 20.1 million are expected in 2100 [3]. In Portugal, the centenarian population was 589 in 2001 [4] and 1,526 in 2011 [5]. These data show that this age group has almost tripled within a decade. Although centenarians still represent a small proportion of the world's total population, the remarkable growth of this age group has generated different studies worldwide.

Centenarian studies and research about longevity have increased considerably in recent decades [6] and are currently recognized as providing important contributions to the understanding of what can be “successful aging.” Through a review of the results of international centenarians' studies, it is possible to determine their main sociodemographic characteristics, health, functionality, and psychosocial features. Within the range of these studies, we can highlight an extensive number of countries that have already profiled their centenarian population, such as Denmark [7, 8], Greece [9], Italy [10], USA [11, 12], Japan [13], Germany [14], Sweden [15], and Australia [16].

Research about centenarians and exceptional longevity contemplates variables pertaining to the aging process, such as its genetic, environmental, biomedical, and psychosocial dimensions [17]; however, aging has assumed a more biomedical perspective rather than a psychosocial one [18]. Centenarians constitute a very heterogeneous age group comprising, on one hand, individuals who live in the community by themselves or with family members and who are cognitively intact and autonomous and, on the other hand, centenarians who have some kind of cognitive impairment and/or are functionally dependent [12, 19–21]. Concerning health and functional capacity, a pronounced variability is also observed [21], but the great majority of studies that compare centenarians with younger groups show an increasing number of difficulties [19] and reveal that physical frailty and chronic conditions are more common at the age of 100+ than in other age groups [20].

In Portugal, the first systematic study about centenarians started in 2015, the PT100 Oporto Centenarian Study [22]. This is a population-based study developed in the metropolitan area of Porto, a seaside area in Northern Portugal, where the main urban centers are located. The well-documented role of the environmental and contextual resources in the aging (e.g., [23, 24]), namely, the distinguishing characteristics of urban versus rural or inland versus seaside environments [25, 26], motivated the development of the present satellite study, the Beira Interior Centenarian Study (PT100-BI). This study closely follows the same study design and methodology of the PT100 [22] and was conducted in the inner part of the country, around the city of Covilhã, in an area with similar geographical extension to that of the Porto metropolitan area.

There is a consensus in literature about the asymmetric socioeconomic reality in Portugal, accentuating the contrast in life conditions and public equipment available in the countryside versus seaside [27]. The concentration of major decision centers and the progressive shutting down of public services in inland Portugal, such as schools, health centers, maternities, and train lines, have accentuated these differences over the years [27]. Inland Portugal is therefore far from the centers of production, consumption, and larger institutions (particularly large hospital centers), being one of the areas with the most aged populations of Portugal, with a low population density, high rate of migration, and small towns and several villages. Most villages do not have any health services or trade and the public transport network to the centers where these services exist is scarce or even nonexistent.

The Portuguese scenario on aging corresponds with the demand for a high level of social and health services specifically designed for elderly people, since Portugal is a country that has only recently incorporated aging questions into its political, social, professional, and scientific agendas. For instance, Portugal is one of a reduced number of European countries where there is not a geriatric specialty among physicians. Profiling the oldest old population and their specific use of services has been a recent concern among Portuguese researchers (e.g., [28, 29]), but further insights are needed from deprived regions like rural areas, which are indicated as areas of increased risk of poor health [25, 30, 31]. According to Santana [32], fragile rural communities had higher mortality rates, higher levels of aging population, lower levels of education, poorer geographical accessibility to health care, and higher alcohol consumption. On the other hand, areas of intensive urbanization, such as Porto, present higher levels of education, geographical proximity to health care, higher incidence rates of AIDS and tuberculosis, and unfavorable general health conditions.

Geographic disparities on the availability of health services are the main obstacle to the unmet medical needs in Portugal, namely, the fact that hospitals located outside large metropolitan areas tend not to offer all medical specialties [33]. Regarding gender differences, in Portugal, it is observed that the avoidable mortality rate through the provision of quality and timely health care is higher in men than in women [33]. Although access to health care is formally identical for both men and women, due to the process of gender typing, women continue to benefit more from formal health care.

Profiling Portuguese individuals who have reached 100 years of age in rural places is thought to be an important step forward in understanding the longevity process in our country, as it can also improve service delivery and programming, particularly when becoming a centenarian may not be a rarity. Furthermore, studying the health status of centenarians from a rural area is important for longevity research as it allows the characterization of a group where the access to the services and health care can be more limited, as is the case in Portugal. This study aims to present sociodemographic information, health conditions, and centenarians' use of services among those who live in Beira Interior and add to the available knowledge in Portugal about this population.

## 2. Participants and Methods

### 2.1. Study Design and Sampling

The participants included in this study were centenarians from Beira Interior, Portugal, and come from a geographical area that includes Beira Interior Norte, Beira Interior Sul, Cova da Beira, and Serra da Estrela (comprising 19 municipalities in total) with an area involving around 9,000 km^2^. This region has 311,051 inhabitants, 27.72% aged 65 and older, and 100 centenarians, according the last National Census. As the data collection reported on centenarians in 2013/14 and the last available census was from 2011, there was a query and search for the location of potential centenarians that, in 2011, were older than 96 years. No exclusion criteria to participate in the study were established other than not being 100 years old (accomplished by age validation procedures, i.e., via confirmation with identity card or birth certificate). This was the only criterion used, as our aim was to have a descriptive profile of all the centenarians in this geographical area. Different levels of participation were, therefore, expected: (i) a basic level (total number of centenarians identified); (ii) those who were not able to participate owing to physical/mental status and/or those who did not want to participate (providing only elementary data); (iii) those who had a partial protocol assessment; (iv) and those who were able to have a full assessment.

The first step for recruiting centenarians was to identify and locate all potential participants in each municipality and parish. This was made based on the census information and through contacting parish councils, local churches, nursing homes, institutions, and health care centers. Then, in the case of centenarians residing in nursing homes, a contact was initially made with the institution's technical director to introduce the study and request collaboration with the research, followed by contacting the centenarians and/or their proxy. As for the centenarians who lived in the community, researchers contacted the centenarians and/or their relatives directly (in some cases the contact was mediated by local research partners who were enrolled in the identification of centenarians: doctors, nurses, social workers, or parish council).

The presentation of the study was always implemented, being guaranteed the understanding of the study and its implications. Informed consent for participating in the study was fully applied. All centenarians (and/or their proxies in case of cognitive impairment) signed a written informed consent form. The study followed all ethical procedures in accordance with the Declaration of Helsinki and was approved by the Ethics Committee of Hospital Sousa Martins, Guarda, Portugal.

A total of 130 potential centenarians were contacted, and of these 29 were excluded. Eight centenarians died between the first contact and the interview, four centenarians refused collaboration due to serious health problems, and five centenarians did not show interest in participating in the study. In four cases, the centenarians or their relatives refused to participate in the study. In eight cases, the reported age could not be proven by the documents/registration. The final sample comprised 101 centenarians.

Information was collected during interviews in one, two, or more sequential sessions. Two researchers of the PT100 Beira Interior team who had been trained with the PT100 Porto team conducted the interviews. Most information was directly obtained from the centenarians. In case of cognitive impairment or in the cases where the centenarian, despite the fact that he/she did not have cognitive impairment, expressed doubts regarding the questions asked (e.g., related to diseases, medication, and use of health services), the information was obtained from the proxies (or complemented with a reference professional in those situations where the centenarian lived in a nursing home).

### 2.2. Instruments

The interview included the use of an extended protocol especially developed in the context of the Oporto Centenarian Study [22]. This protocol covered a wide range of measures and instruments: sociodemographic characteristics of the respondents, overall health status, lifestyle, relationships, psychological variables (e.g., personality and wellbeing), anthropometric measures, and service use. For this particular study, we have only considered sociodemographic information, health status (functionality and cognition), and health and social services use.

Regarding sociodemographic data, information was obtained on age, sex, marital status, schooling, type of accommodation, and whether the centenarian had ever lived outside Portugal. Information about centenarians' income was also collected, namely, the main source of income, monthly value, income management, and whether it was enough to cover regular and health related expenses (i.e., income adequacy).

The morbidity profile was assessed using a self-report checklist of diseases provided by OARS—the Multidimensional Functional Assessment Questionnaire [34] adapted for Portuguese population [35]. The checklist included the following health conditions: high blood pressure, heart condition, diabetes, chronic lung disease, stomach ulcers, irritable bowel syndrome or other serious problems with stomach or bowels, cirrhosis or any other serious liver problems, problems with kidneys, frequent urinary infections, urinary incontinence, prostate problems (for men), problems with vision, problems with hearing, arthritis (hands, knee, hip, shoulder, and spine), and osteoporosis at the time of the interview.

As for cognitive status, the Global Deterioration Scale (GDS) [36] provided an overview of the stages of cognitive function for those suffering from a primary degenerative dementia, such as Alzheimer's disease. GDS is an interviewer rating of subjective memory complaints, orientation, and functional ability covering seven stages of deterioration. The interviewers according to the information collected and the observation of the centenarians' behavioral characteristics completed this scale. Each participant was rated according to seven distinct stages: (1) no subjective complaints of memory deficit; (2) subjective complaints of memory deficit; (3) earliest clear-cut deficits, with evidence of memory deficit in an intensive interview; (4) clear-cut deficit on clinical interview with decreased knowledge of current and recent events; (5) patient being no longer able to survive without assistance and being unable to recall major relevant aspects of their current lives; (6) occasionally forgetting the name of the spouse upon whom they are dependent, retaining some knowledge of their past lives, and requiring some assistance with activities of daily living; and (7) very poor cognitive skills with all verbal abilities being lost. Stage 1 (no cognitive decline) and stage 2 (very mild cognitive decline) were considered as indicative of good cognitive functioning.

The use of the health services was assessed by self-report questions on the number of visits to the family doctor, number of visits to the emergency service, and number of hospitalizations during the preceding year. The number of drugs that centenarians took per day was also considered. The use of support services was assessed by a self-report list on which it was marked if the centenarian did or did not use each service. The list included day center, social center, house support service, outpatient care nursing, nursing, physiotherapy, speech therapy, and occupational therapy. There was the possibility to indicate other types of services. The use of technical aids was explored by means of a list of the most frequently used technical aids by older population: walking stick, hiker, wheelchair, hearing aid, glasses, and the possibility to indicate other types of services. Interviewers complementarily classified the centenarians' mobility in one of three categories: (1) limited to a wheelchair or bed; (2) able to get out of the chair or the bed but does not; and (3) gets out.

### 2.3. Statistical Analysis

The statistical analysis was conducted using the Statistical Package for the Social Sciences (SPSS) version 21. Descriptive analysis included assessment of frequency distributions and the calculation of measures of central tendency and dispersion. Sex differences were considered based on their clinical and psychosocial relevance. Statistical significance of the observed sex differences was carried out using the* chi-square test* and* Student's t-test*. The level of statistical significance used was* p* < 0.05.

## 3. Results

A total of 101 centenarians participated in this study: 14 (13.9%) males and 87 (86.1%) females. The mean age of the centenarians was 101.1 years (SD = 1.5), and the median age was 101. The age range was 100–108 years. Most centenarians were widows (91.1%); 5.9% of the participants, all women, were single, and 3% of the participants (two men and one woman) had partners who were still alive. The differences between men and women concerning marital status are statistically significant, with more widowers and never-married women and fewer women who had married when compared to men.

More than half of the centenarians never attended school (53.4%), and the mean of school years attended was 1.45 (SD = 1.97). Men reported significantly more years of education than women. As for the type of accommodation, 50 centenarians (49.5%) lived in institutions, 47 (46.6%) lived in houses/flats, and 4 (4%) lived in other kinds of accommodation (such as pensions, religious institutions, or house annexes). More women (51.7%) than men (35.7%) were found to be living in nursing homes; however, this difference was not statistically significant. Of the participants, 86 (85.1%) always lived in the same region they currently live in, showing no significant geographical mobility. Only the centenarians who reported having been emigrants (15, 11.5%) lived in a different region than Beira Interior. More men than women reported having been emigrants and this difference was statistically significant (see Table 1).

Concerning monthly income, the main resource of 68 centenarians (67.3%) was a state pension, followed by social security for 30 centenarians (29.7%). Statistically significant differences were observed on this aspect, with more women (71.3%) than men (42.9%) reporting that their main source of income was their pension and more men (50%) than women (26.4%) being dependent on social security. The income per month for 18 centenarians (17.8%) was less than €250; for 66 centenarians (71%), it was between €250 and €500 and for nine (9.7%) of them it was more than €500 per month. 40 centenarians (39.6%) had difficulties in paying their expenses, and 43 centenarians (42.6%) could only cover the most essential needs.

We observed statistically significant differences between women and men regarding income management: there was a higher percentage of women (45.9%) than men (21.4%) who considered the income just manageable to get by on and a higher percentage of men (42.9%) than women (39%) who struggled to make ends meet. Only five male centenarians (35.7%) and seven female centenarians (8%) stated that not only was the money enough but also they could save a little extra. Concerning income adequacy for medical expenses specifically, 17 (16.8%) did not report difficulties, 33 (32.7%) indicated it was not very difficult, and 46 (45.5%) reported difficulties. No statistically significant differences were observed between men and women (see Table 2).

Table 3 presents the prevalence of self-reported health problems. More than half of the centenarians reported not having had health problems related to high blood pressure, a heart condition, diabetes, chronic lung disease, stomach ulcers, irritable bowel syndrome, or other serious problems with stomach or bowels, cirrhosis or any other serious liver problem, problems with kidneys, frequent urinary infections, urinary incontinence, prostate problems, arthritis, or osteoporosis. In the diseases checklist, the most prevalent diseases were urinary incontinence (32 cases, 31.7%) followed by high blood pressure (24 cases, 23.8%) and heart conditions (20 cases, 19.8%). Vision and hearing impairments presented a much higher incidence compared to the other health problems. There were 61 cases (50 women and 11 men) that reported suffering from problems with vision and 74 cases (11 men and 63 women) that reported hearing problems. The analysis of sex differences only indicates statistically significant differences with respect to arthritis, with 19 women (21.8%) and no men having this health problem. It is worth mentioning that 39 centenarians (38.6%) reported never having had a serious health problem throughout their lives.

Concerning the general cognitive status of the centenarians, 48 participants (47.5%) presented no cognitive impairment, 32 (10.9%) presented earlier clear-cut deficits, and 40 (39.5%) had a level of cognitive impairment that invalidated their functioning. There are no statistically significant sex differences (see Table 4). As for the centenarians' mobility, 39 (38.6%) were confined to a wheelchair or bed, 11 (10.9%) were able to get out of the chair or bed but did not do so, and 51 (50.5%) had clear autonomy of movement.

With regard to the use of health services, several centenarians had no assisting doctor (see Table 5). For those who did, the annual average use was 1.09 (±1.655). On average, women go to the doctor more often (1.13 ± 1.731) than men (0.86 ± 1.167); however, this difference was not statistically significant. Concerning the use of the emergency service, 67 centenarians (66.3%) had not used this service in the last year. Men had a statistically significant higher mean use of this service (0.71 ± 0.825) than women (0.31 ± 0.562). Concerning hospitalizations, 90 centenarians (89.1%) reported not having been hospitalized. The mean of these centenarians' hospitalizations in the last year was 0.33 (±1.891), and there were no statistically significant sex differences.

The mean number of daily drugs taken was 2.87 (±2.455). There were 16 centenarians (2 males and 14 females) taking no medication, 44 (43.5%) taking between 1 and 3 drugs, and 35 (34.7%) taking more than 3 drugs. There were no sex differences concerning the mean number of drugs and the number of drugs used by these centenarians per day.

The support services for elder people are scarcely used by the centenarians from Beira Interior (see Table 6). The most widely used service was nursing (*n* = 29, 28.7%), followed by house support services (*n* = 13, 12.8%) and day centers (*n* = 8, 7.9%). Social centers, speech therapy, outpatient nursing care, physiotherapy, speech therapy, occupational therapy, and other kinds of services had a percentage of use equal to or less than 4%. There were no sex differences concerning the use of support services by centenarians in this geographical area.

Among the available technical aids, 29 centenarians (28.7%) used walking sticks, 13 (12.9%) used hikers, and 37 (36.6%) used wheelchairs. The use of glasses stands out, as they are the most used technical aid: 38 centenarians (37.6%) need them. Hearing aids were used by only 3 (3%) of the centenarians (see Table 6).

## 4. Discussion and Conclusions

This is one of the few studies about Portuguese centenarians and it focuses on a specific geographic population living in the countryside. It provides information about the sociodemographic profile of 101 centenarians living in one of the regions with the largest aging population in Portugal, and it provides valuable information about their health profiles and their use of the health support system, services, and technical aids.

With regard to the sociodemographic profile of this group, as expected, women outnumber men and the male/female ratio was 6.1:1 (87 women/14 men). Women represented 86.1% of the centenarians, which is in accordance with the values reported by other studies on centenarians (e.g., [30]) and with the profile of the overall Portuguese centenarian population [29] and of the Oporto centenarian study [22]. More than half of the centenarians (53.4%) never attended school. This is a surprisingly positive number if we take into account the fact that, in 1900, about ten years before most of the centenarians enrolled in this study were born, 75% of the population was illiterate [37], so it could be expected that a higher rate of centenarians would similarly be illiterate, especially in a rural area, where traditionally people had fewer chances of attending school due to restrictions to education access and demanding agricultural chores in childhood.

In the current study, sex differences were found in some sociodemographic characteristics. There are more widowers and never-married women than men, which may be related to the much higher survival rate of women in older age compared to that of men and to the fact that women tend to marry men who are older [38]. Concerning education, men reported a significantly higher average number of years of education than women. This is expected and reflects the dominant position of men in general Portuguese society from 100 years ago. In these centenarians' generation, especially in rural areas, the illiteracy rate was higher and more prevalent among women, as their social roles were mainly connected to the family and home caring [37]. Sex differences are also apparent in the higher number of men having been emigrants, which could be related to the fact that a man's role was essentially that of a provider. Portugal has a long and important history of emigration to different countries in the world, more evident in rural and inland areas of the country, where living conditions were worse, compelling men to leave the country first. This fact was also confirmed in our study.

Concerning centenarians' income, the majority had a monthly income between €250 and 500. This value is rooted in the Portuguese sociohistorical background of a dictatorship that was characterized by a global poverty [22] and in the fact that most of these centenarians worked in agriculture with low earnings. With these incomes, the majority showed difficulties in paying their expenses, namely, health treatments that imply costly travel arrangements to hospital centers located away for their living geographical area. This can help to explain the reduced use of health care services and technical aids among these centenarians.

With regard to the type of accommodation, the number of centenarians living in private households (50.6%) is lower than the national rate of 71% [29]. This may be due to the fact that most of them live in very small isolated areas with a very reduced number of inhabitants, without proper housing conditions, and with a reduced use of technical support and assistance services (e.g., domiciliary care) to enable them to live in their homes. Another explanation is that the rural areas of inland Portugal had in the 1960s and 1970s a strong migratory movement to other European countries [37]. For this reason, many of these centenarians' children live abroad and therefore cannot be their caregivers, which, in turn, has led them to recur to nursing homes.

Regarding diseases, back in the beginning of last century, about the time the people in this study were born, the rate of Portuguese infant mortality was 140‰ [32]. During their childhood and adult life, there were no vaccinations or health care support for diseases that are now treatable, which means that many of these centenarians resisted several pathologies in very adverse circumstances, rendering them survivors and the most resistant of their generation. This high percentage of healthy centenarians supports Evert et al.'s perspective that exceptional longevity can be related to one's ability to escape all major age-related diseases [37]. According to this perspective, this group of centenarians could be labelled as “escapers” (i.e., individuals with no diagnosis of major age-related diseases up to 100 years of age), which could suggest the existence of specific conditions that allowed the survival of the most robust individuals [39]. On the other hand, we can also hypothesize that better health care access in inner part of the country can lead to a higher number of centenarians in this rural area, probably, with increased levels of morbidity, according to Robine et al.'s (2010) perspective about the trade-off between the level of mortality selection and the functional health status of the oldest old survivors. This perspective could explain the fact that centenarians from Beira Interior show lower prevalence of diseases when compared to global results of age-related illnesses for the centenarians from Porto (with easier access to health care).

In the context of reported diseases, the high rate of sensory problems connected to vision (60.4%) must be highlighted: while glasses are the most used technical aid mentioned by centenarians, only 38 (37.6%) used them. Similarly, problems with hearing are mentioned by 73% of the centenarians; nevertheless, hearing aids were used by only 3%. This data indicates a low use of technical aids by this group, a finding that can be connected to lack of information, low incomes, and difficulties in income management or, eventually, ageist attitudes in the potential treatment of these sensorial gaps with technical aids, in line with findings observed in the Oporto Centenarian Study [40]. On the other side, this data indicates that there is a possibility for compensation or attenuation of part of the potential sensorial gaps of these centenarians, which could have important implications for cognitive performance, interpersonal relationships, and overall quality of life.

As for the general cognitive status of the centenarians, nearly a half (47.5%) presented no cognitive impairment (GDS1 and GDS2), which is slightly above the values observed in the Oporto Centenarians (30,3%) [22]. It is very difficult to compare these results with results from other international studies because the cognitive status was differently evaluated. Even considering this important methodological limitation, some assumptions can be made. For instance, it is possible to relate Beira Interior centenarians' cognitive status with their isolation, as this may have enhanced their ability to adapt and to find solutions. Comparing the prevalence of dementia obtained in this study with values observed in a review on cognition in centenarians made by Ishioka and Gondo [41], it is in the range found, which is from 33% to 100% (average was 62%, 48,5% for males and 66,1% for females). Comparing our results to specific centenarian studies, we can cautiously conclude that, due to the mentioned methodological concerns, the centenarians of Beira Interior present lower prevalence of dementia than in Canadian centenarians [42], Danish centenarians [19], Dutch centenarians [43, 44], Korean Centenarian [45], Italian centenarians [46], Japanese centenarians [47–49], and centenarians from the Georgia Centenarian Study [12]. On the other hand, the prevalence of dementia in Portuguese centenarians of Beira Interior is higher than that in Finish centenarians [50]. In this specific group of Portuguese centenarians from Beira Interior, there are no statistically significant differences between men and women in cognition, which contradicts results from other centenarians studies (e.g., [46, 48]) that pointed out dementia prevalence rates as being higher among women than among men.

With regard to the use of the health services, the annual average use of the assistant doctor is notably under the values observed in other centenarian studies (e.g., [9, 19, 42, 51]) and also below the average of consultations of the Portuguese population, which is near 4 consultations per year [52]. Nonetheless, the data confirms the importance of a primary health care physician for this group, to whom most Portuguese centenarians have access, even though the number of appointments is reduced. The data does not include information about access and the number of appointments with medical specialists, which would have been important in order to discuss and analyze their role in centenarian caretaking. Considering the rural context the centenarians are living in and the difficulty in accessing health services, home care and other strategies would mean a significant improvement in the use of primary health care and avoiding emergency services.

Finally, with regard to hospitalizations, 90% of centenarians reported not having been hospitalized. This result is specifically the reverse of that found in other studies, such as Andersen-Ranberg et al.'s [19] in Denmark, which refers to 94% hospitalizations. On the other hand, it is a very similar result to that of Rochon et al. [42], in Canada, which indicates 18.2% hospitalizations in the last year. In fact, there are contradictory facts in the literature concerning the higher rates of hospitalizations in advanced age. There is evidence that the oldest old have higher rates of hospitalizations [53], although hospital admissions are higher in nonagenarians than in centenarians [54] and there are a lower number of hospital admissions in centenarians than in persons who died before this age [8].

Concerning support services to elderly people, these were scarcely used by these centenarians from rural areas. Data reveal a very low use of services and equipment needs other than technical interventions. The very low level of use of interventions like psychological support or physiotherapy may be associated with the reduced offer of such services in the geographical area and/or with low (health) literacy levels that could be constraining the recognition of their benefits to the centenarians. Moreover, ageist attitudes towards such benefits should also be appointed as potential justifications. Nevertheless, referring this group to the health services mentioned could make up for the deficits and problems identified and improve their dignity and life quality.

This study has several limitations, namely, as mentioned before, the fact that it was mostly based on self-reporting measures, which may have led to an underestimation of diseases and service uses. Though important, self-reported data should be crosschecked with objective health measures and data connected to the use of health services. The most important contribution of this study consists in the description of a centenarian Portuguese population resident in a rural environment. The profile obtained will help to better understand health care service use (and needs) by this population in order to promote life quality of centenarians in contexts with low population density and sparse health care services. Further research is needed to understand the role of contextual variables in the Beira Interior centenarians' adaptive process to advanced longevity. The comparative analysis of this group with other centenarian groups will also be an important step in understanding the aspects that differentiate centenarians living in a region that is predominantly rural.

## Figures and Tables

**Table 1:** Sociodemographic profile of the Beira Interior Portuguese centenarians (*N* = 101) by sex.

		**Male**	**Female**	**Total**	***p***
**Sex**	Female	14 (13.9%)	87 (86.1%)	101	

**Age (in years)**					0.576
	Mean (±SD)	100.93 ± 1.439	101.17 ± 1.519	101.14 ± 1.504	
	Median	100.50	101	101	
	Range (min/max)	100–105	100–108	100–108	

**Marital status**	Never married	0	6 (6.9%)	6 (5.9%)	0.018
	Married	2 (14.3%)	1 (1.1%)	3 (3%)	
	Widowed	12 (85.7%)	80 (92%)	92 (91.1%)	

**Did you attend school?**	No	5 (35.7%)	42 (48.3%)	47 (46.5%)	0.617
	No, but can read and write	1 (7.1%)	6 (6.9%)	7 (6.9%)	
	Yes	8 (57,1%)	37 (42.5%)	45 (44.6%)	
	N/A	-	2 (2.3%)	2 (2%)	

**Years of education**					0.044
	Mean (±SD)	2.46 (± 2.757)	1.28 (± 1.775)	1.45 (± 1.969)	
	Median	3	0	0	
	Range (min/max)	0/9	0/7	0/9	

**Type of accommodation**	House/flat	8 (57.1%)	39 (44.8%)	47 (46.6%)	0.637
	Nursing home	5 (35.7%)	45 (51.7%)	50 (49.5%)	
	Other	1 (7.1%)	3 (3.4%)	4 (4%)	

**Residence outside Portugal**	No	9 (64.3%)	77 (88.5%)	86 (85.1%)	0.033
	Yes	5 (35.7%)	10 (11.5%)	15 (14.9%)	

**Table 2:** Income of the Beira Interior Portuguese centenarians (*N* = 101).

		**Male**	**Female**	**Total**	***p***
		**(** **n** ** = 14)**	**(** **n** ** = 87)**		
**Main source of income**	Pension	6 (42.9%)	62 (71.3%)	68 (67.3%)	0.008
	Social security	7 (50%)	23 (26.4%)	30 (29.7%)	
	Financial aid by children	1 (7.1%)	0	1 (1%)	
	N/A	-	2 (2.3%)	2 (2%)	

**Income per month**	<€250	1 (7.1%)	17 (19.5%)	18 (17.8%)	0.243
	€250–500	10 (71.4%)	56 (64.4%)	66 (65.3%)	
	€500–750	2 (14.3%)	5 (5.8%)	7 (6.9%)	
	€750–1,000	1 (7.1%)	1 (1.1%)	2 (2%)	
	N/A	-	8 (9.2%)	8 (7.9%)	

**Income management**	You cannot make ends meet	6 (42.9%)	34 (39%)	40 (39.6%)	0.021
	You just manage to get by	3 (21.4%)	40 (45.9%)	43 (42.6%)	
	You have enough money with a little extra	5 (35.7%)	7 (8%)	12 (11.9%)	
	Money is not a problem	0	3 (3.4%)	3 (3%)	
	N/A	-	3 (3.4%)	3 (3%)	

**Cover medical expenses**	Not difficult at all	4 (28.6%)	13 (14.9%)	17 (16.8%)	0.566
	Not very difficult	5 (35.7%)	28 (32.2%)	33 (32.7%)	
	Somewhat difficult	4 (28.6%)	26 (29.9%)	30 (29.7%)	
	Very difficult	1 (7.1%)	15 (17.2%)	16 (15.8%)	
	N/A	-	5 (5.7%)	5 (5%)	

**Table 3:** Current health problems of the centenarians of Beira Interior (*N* = 101).

	**Male**	**Female**	**Total**	***p***
	**(** **n** ** = 14)**	**(** **n** ** = 87)**		
**High blood pressure**	5 (35.7%)	19 (21.8%)	24 (23.8%)	0.209

**Heart condition**	5 (35.7%)	15 (17.2%)	20 (19.8%)	0.109

**Diabetes**	1 (7.1%)	3 (3.4%)	4 (4%)	0.455

**Chronic lung disease**	3 (21.4%)	11 (12.6%)	14 (13.9%)	0.301

**Stomach ulcers, irritable bowel syndrome, or other serious problems with stomach or bowels**	4 (28.6%)	7 (8%)	11 (36,6%)	0.044

**Cirrhosis or any other serious liver problems**	0	1	1 (1%)	0.861

**Problems with kidneys**	0	2 (2.3%)	2 (2%)	0.741

**Frequent urinary infections**	0	4 (4.6%)	4 (4%)	0.545

**Urinary incontinence**	5 (35.7%)	27 (31%)	32 (31.7%)	0.473

**Prostate problems (for men)**	3 (21.4%)	-	-	

**Problems with vision**	11 (78.6%)	50 (57.5%)	61 (60.4%)	0.113

**Problems with hearing**	11 (78.6%)	63 (72.4%)	74 (73.3%)	0.452

**Arthritis (hands, knee, hip, shoulder, and spine)**	0	19 (21.8%)	19 (18.2%)	0.043

**Osteoporosis**	0	8 (9.2%)	8 (7.9%)	0.289

**Other medical conditions**	1 (7.1%)	8 (9.1%)	9 (9.9%)	0.637

**Table 4:** Global Deterioration Scale of the Portuguese centenarians from Beira Interior (*N* = 101).

****	**Male**	**Female**	**Total**	***p***
	**(** **n** ** =14)**	**(** **n** ** = 87)**		
**No subjective complaints of memory deficit**	2 (14.3%)	8 (9.2%)	10 (9.9%)	0.446
**Subjective complaints of memory deficit**	5 (35.7%)	33 (37.9%)	38 (37.6%)	
**Earlier clear-cut deficits**	3 (21.4%)	8 (9.2%)	11 (10.9%)	
**Clear-cut deficit in clinical interview**	2 (14.3%)	6 (6.9%)	8 (7.9%)	
**Patient can no longer survive without assistance**	0	7 (8%)	7 (6.9%)	
**Occasionally forgets the name of the spouse upon whom they are dependent **	0	12 (13.8%)	12 (11.8%)	
**All verbal abilities are lost **	2 (14.3%)	11 (12.6%)	13 (12.9%)	
**N/A**	0	2 (2.3%)	2 (2%)	

**Table 5:** Use of health services/support by the Portuguese centenarians from Beira Interior throughout the last year (*N* = 101).

	**Male**	**Female**	**Total**	***p***
**Family doctor**				0.575
Mean (±SD)	0.86 (±1.167)	1.13 (±1.731)	1.09 (±1.655)	
Median	0.50	0.00	0.00	
Range (min/max)	0/4	0/9	0/9	

**Emergency services**				0.023
Mean (±SD)	0.71 (±0.825)	0.31 (±0.562)	0.37 (±0.620)	
Median	0.50	0.00	0.00	
Range (min/max)	0/2	0/2	0/2	

**Hospital/continuous care**				0.925
Mean (±SD)	0.29 (±0.611)	0.34 (±2.032)	0.33 (±1.891)	
Median	0.00	0.00	0.00	
Range (min/max)	0/2	0/15	0/15	

**Table 6:** Use of elder support services and technical aids by the Portuguese centenarians from Beira Interior (*N* = 101).

		**Male (** **N** ** = 14)**	**Female (** **N** ** = 87)**	**Total**	***p***
**Day center**	No	14 (100%)	77 (88.5%)	91 (90.1%)	0.281
****	Yes	0	8 (9.2%)	8 (7.9%)	
	N/A	0	2 (2.3%)	2 (2%)	

**Social center**	No	14 (100%)	80 (91.9%)	94 (93.1%)	0.623
****	Yes	0	3 (3.4%)	3 (3%)	
	N/A	0	4 (4.6%)	4 (4%)	

**House support service**	No	12 (85.7%)	85 (97.7%)	85 (84.2%)	0.589
****	Yes	2 (14.3%)	13 (14.9%)	13 (12.8%)	
	N/A	0	3 (3.4%)	3 (3%)	

**Outpatient nursing care**	No	14 (100%)	81 (93.1%)	95 (94.1%)	0.731
****	Yes	0	2 (2.3%)	2 (2%)	
	N/A	0	4 (4.6%)	4 (4%)	

**Nursing**	No	10 (71.4%)	59 (67.8%)	69 (68.3%)	0.601
****	Yes	4 (4.6%)	25 (28.7%)	29 (28.7%)	
	N/A	0	3 (3.4%)	3 (3%)	

**Physiotherapy**	No	14 (100%)	76 (87.4%)	90 (89.1%)	0.323
****	Yes	0	7 (8%)	7 (6.9%)	
	N/A	0	4 (4.6%)	4 (4%)	

**Speech therapy**	No	14 (100%)	83 (95.4%)	97 (96%)	-
	Yes	0	0	0	
	N/A	0	4 (4.6%)	4 (4%)	

**Occupational therapy**	No	14 (100%)	82 (94.2%)	96 (95%)	0.733
	Yes	0	2 (2.3%)	2 (2%)	
	N/A	0	3 (3.4%)	3 (3%)	

**Other services**	No	13 (92.8%)	74 (85%)	87 (86.1%)	0.402
****	Yes	1 (7.2%)	2 (2.3%)	3 (3%)	
	N/A	0	11 (12.6%)	11 (10.9%)	

		**Technical aids**			

**Walking stick**	No	6 (42.8%)	60 (69%)	66 (65.3%)	0.111
	Yes	6 (42.8%)	23 (26.4%)	29 (28.7%)	
	N/A	2 (14.3%)	4 (4.6%)	6 (5.9%)	

**Hiker**	No	12 (85.7%)	71 (81.6%)	83 (82.2%)	0.155
	Yes	0	13 (14.9%)	13 (12.9%)	
	N/A	2 (14.3%)	3 (3.5%)	5 (4.9%)	

**Wheelchair**	No	9 (64.3%)	52 (59.8%)	61 (60.4%)	0.409
	Yes	4 (28.6%)	33 (33.9%)	37 (36.6%)	
	N/A	1 (7.1%)	2 (2.3%)	3 (3%)	

**Hearing aids**	No	12 (85.7%)	80 (91.9%)	92 (91.1%)	0.360
	Yes	1 (7.1%)	2 (2.3%)	3 (3%)	
	N/A	1 (7.1%)	5 (5.7%)	6 (5.9%)	

**Glasses**	No	6 (42.8%)	52 (59.8%)	58 (57.4%)	0.203
	Yes	7 (50%)	31 (35.6%)	38 (37.6%)	
	N/A	1 (7.1%)	4 (4.6%)	5 (5%)	
